# Supplementary material for: Functional repertoire convergence of distantly related eukaryotic plankton lineages abundant in the sunlit ocean
Source: Cell Genom. 2022 Apr 28;2(5):100123. doi: 10.1016/j.xgen.2022.100123 (PMC9903769; doi:10.1016/j.xgen.2022.100123)
Supplement: Document S1. Figures S1–S21 and Methods S1 [file mmc1.pdf]

Cell Genomics, Volume *2*

Supplemental information

Functional repertoire convergence of distantly related eukaryotic plankton lineages abundant in the sunlit ocean

Tom O. Delmont, Morgan Gaia, Damien D. Hinsinger, Paul Frémont, Chiara Vanni, Antonio Fernandez-Guerra, A. Murat Eren, Artem Kourlaiev, Leo d'Agata, Quentin Clayssen, Emilie Villar, Karine Labadie, Corinne Cruaud, Julie Poulain, Corinne Da Silva, Marc Wessner, Benjamin Noel, Jean-Marc Aury, Tara Oceans Coordinators, Colomban de Vargas, Chris Bowler, Eric Karsenti, Eric Pelletier, Patrick Wincker, and Olivier Jaillon

Supplemental figures

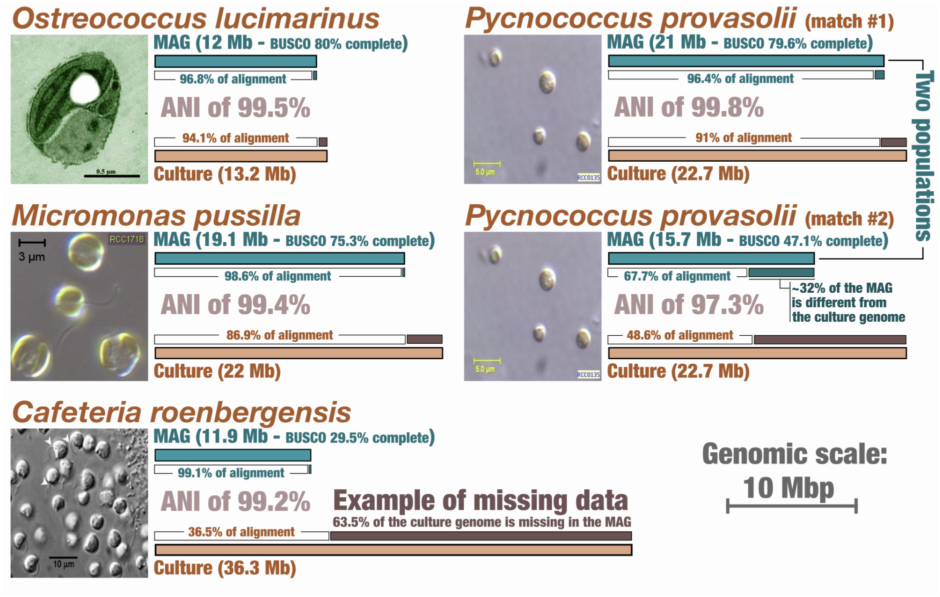

Figure S1. Genomic comparison of *Tara* Oceans MAGs and genomes from culture, Related to Figure 2. The figure summarizes the Average Nucleotide Identity (ANI) and percentage of genomic alignment for five matches between a MAG and a standard culture genome.

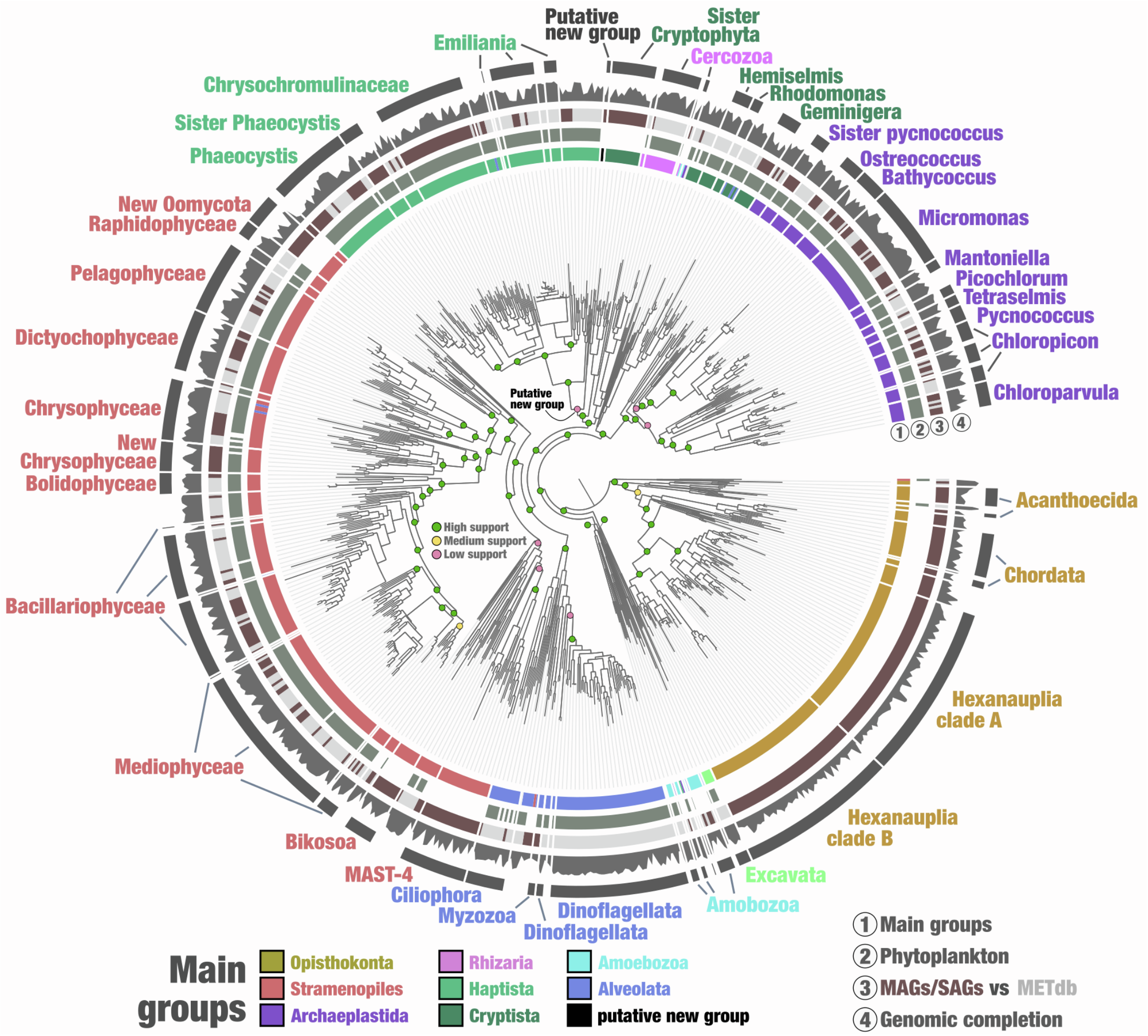

Figure S2. Phylogenomic analysis of the protein sequences of 255 BUSCO genes markers from eukaryotic plankton, Related to Figure 2. The maximum-likelihood phylogenomic tree of the BUSCO gene markers (255 genes) included *Tara* Oceans MAGs and METdb transcriptomes (minimum of 25% of completion) and was generated using a total of 19,785 sites in the alignment and LG+F+R10 model; Opisthokonta was used as the outgroup. The tree was decorated with additional layers using the anvi'o interface. Branches and names in red correspond to lineages lacking representatives in METdb.

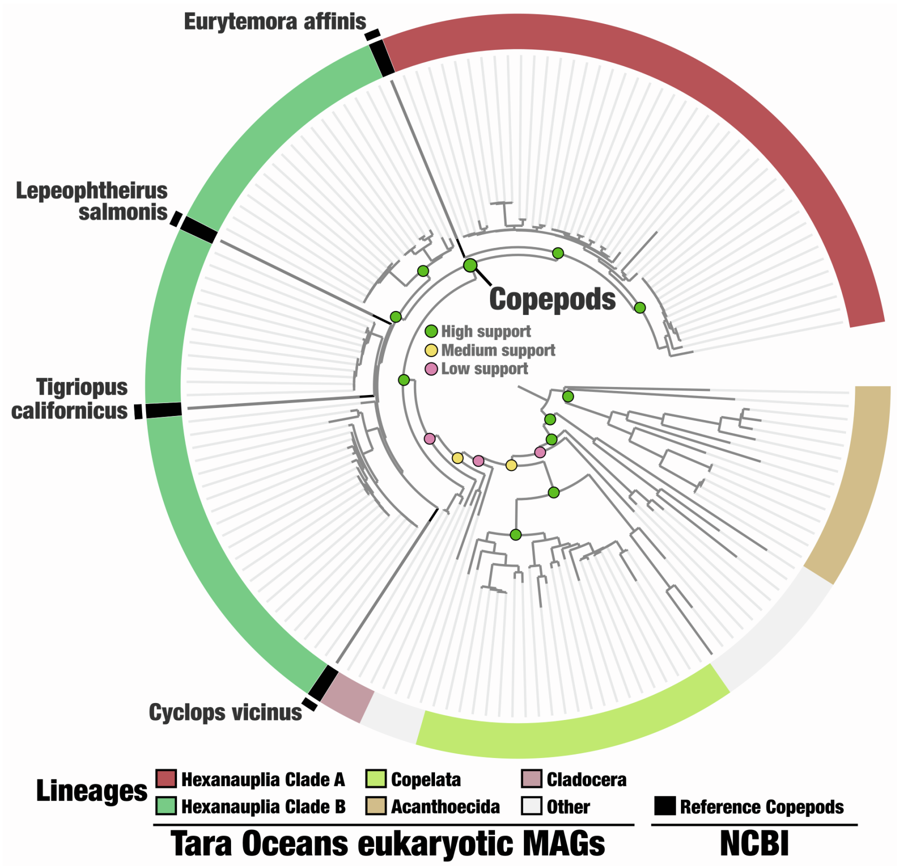

Figure S3: Phylogenetic analysis of concatenated DNA-dependent RNA polymerase II protein sequences from Opisthokonta MAGs and four copepod species, Related to Figure 2. The maximum-likelihood phylogenetic tree of the concatenated two largest subunits of the DNA-dependent RNA polymerases II (two genes) included *Tara* Oceans MAGs and reference copepod genomes (source: NCBI) and was generated using a total of 2,112 sites in the alignment and LG+R4 model (determined by ModelFinder); Acanthoecida were used as the outgroup. Supports for selected clades are displayed. Phylogenetic supports were considered high (aLRT>=80 and UFBoot>=95), medium (aLRT>=80 or UFBoot>=95) or low (aLRT<80 and UFBoot<95) (see Methods).

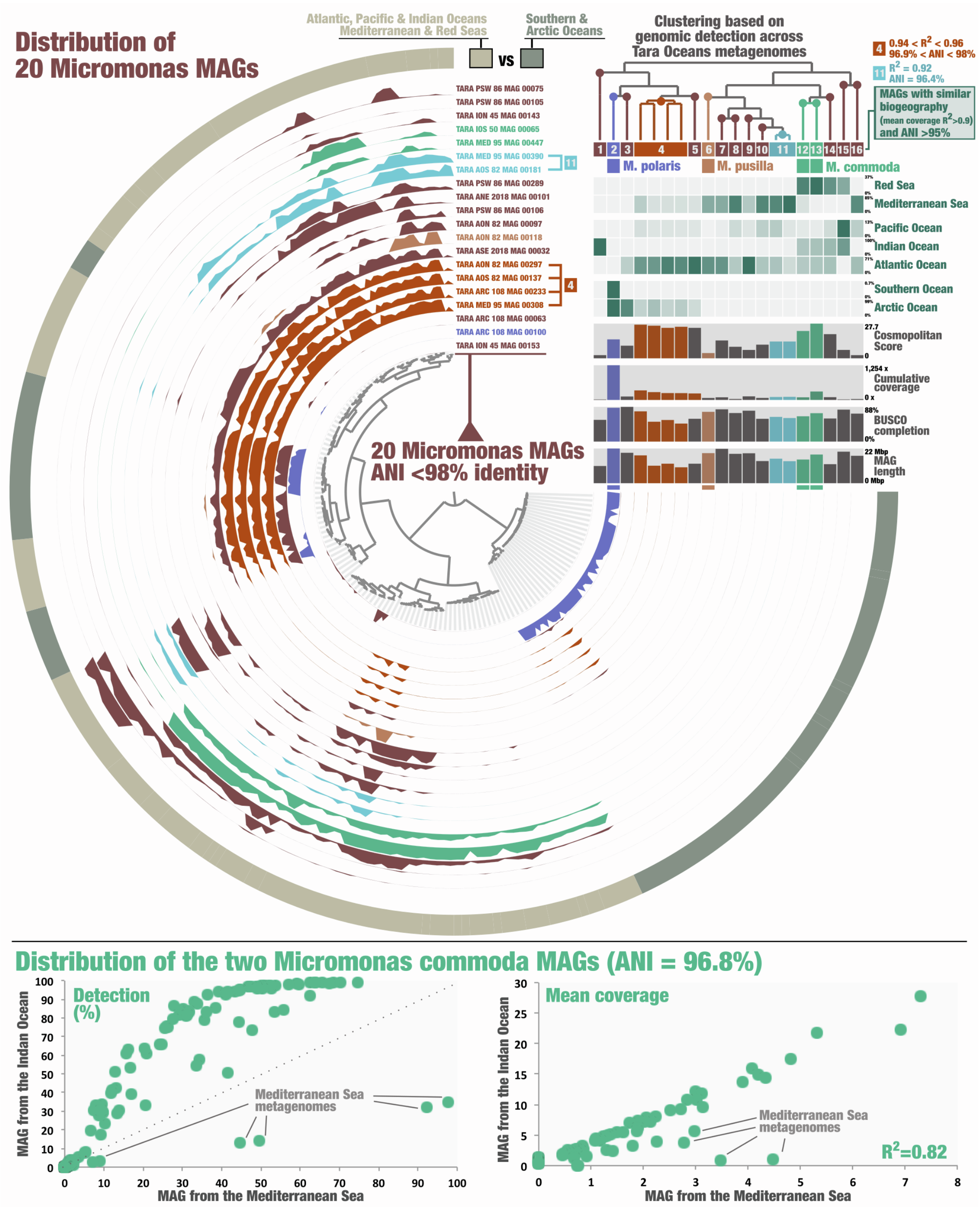

Figure S4: Biogeography of Micromonas populations, Related to Figure 2. Top panel displays the detection of 20 Micromonas MAGs across 258 *Tara* Oceans metagenomes for which at least one MAG was detected. The inner tree organizes the metagenomes as a function of the detection signal, and the tree on the top right corner organizes the MAGs based on the same signal. Thus, MAGs are organized based on similarities in their biogeography. MAGs with a coefficient of determination (R2) > 0.9 for the mean coverage values across metagenomes and average nucleotide identity >95% were linked to the same population Id. Populations "4" and "11" are represented by 4 MAGs and 2 MAGs, respectively. Bottom panel displays the detection (horizontal coverage) and mean coverage of two MAGs affiliated to *Micromonas commoda* across 939 Tara Oceans metagenomes.

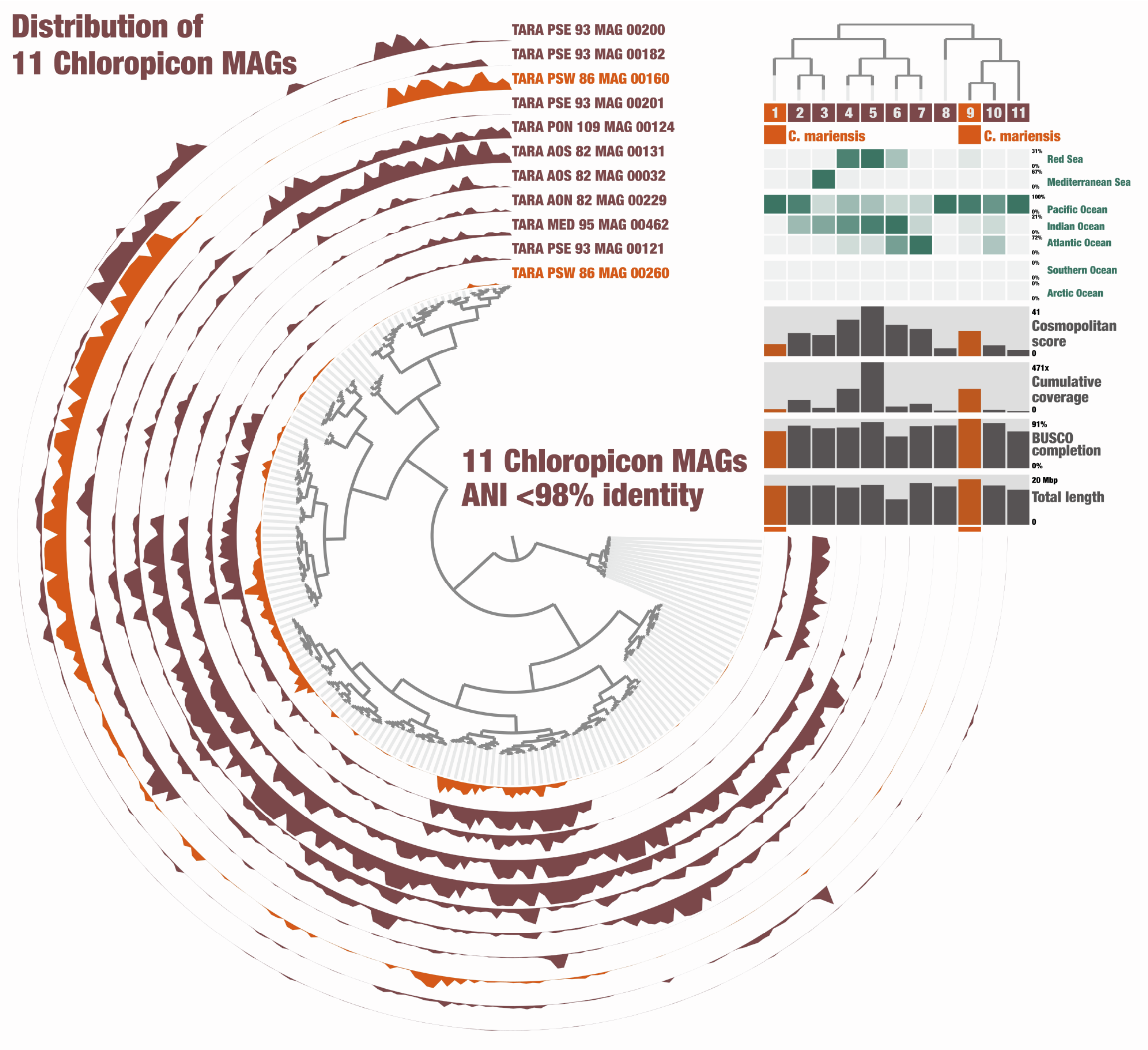

Figure S5: Biogeography of Chloropicon populations, Related to Figure 2. The figure displays the detection of 11 Chloropicon MAGs across 323 *Tara* Oceans metagenomes for which at least one MAG was detected. The inner tree organizes the metagenomes as a function of the detection signal, and the tree on the top right corner organizes the MAGs based on the same signal. Thus, MAGs are organized based on similarities in their biogeography. There were no MAGs with a coefficient of determination (R2) > 0.9 for the mean coverage values across metagenomes and average nucleotide identity >95%. Thus, each MAG was linked to a distinct population Id.

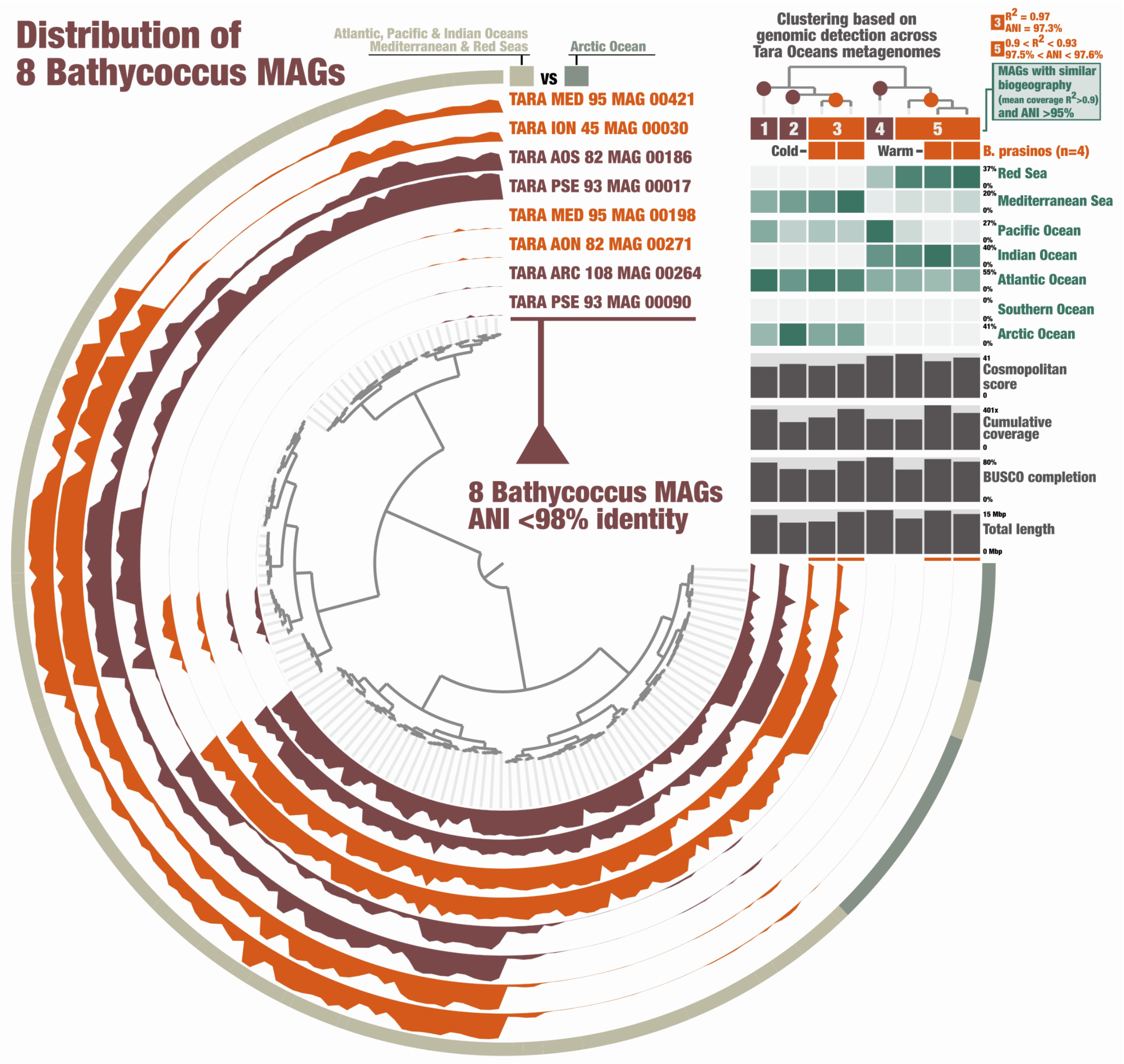

Figure S6: Biogeography of Bathycoccus populations, Related to Figure 2. The figure displays the detection of 8 Bathycoccus MAGs across 231 *Tara* Oceans metagenomes for which at least one MAG was detected. The inner tree organizes the metagenomes as a function of the detection signal, and the tree on the top right corner organizes the MAGs based on the same signal. Thus, MAGs are organized based on similarities in their biogeography. MAGs with a coefficient of determination (R2) > 0.9 for the mean coverage values across metagenomes and average nucleotide identity >95% were linked to the same population Id. Populations “3” and “5” are represented by 2 MAGs and 3 MAGs, respectively.

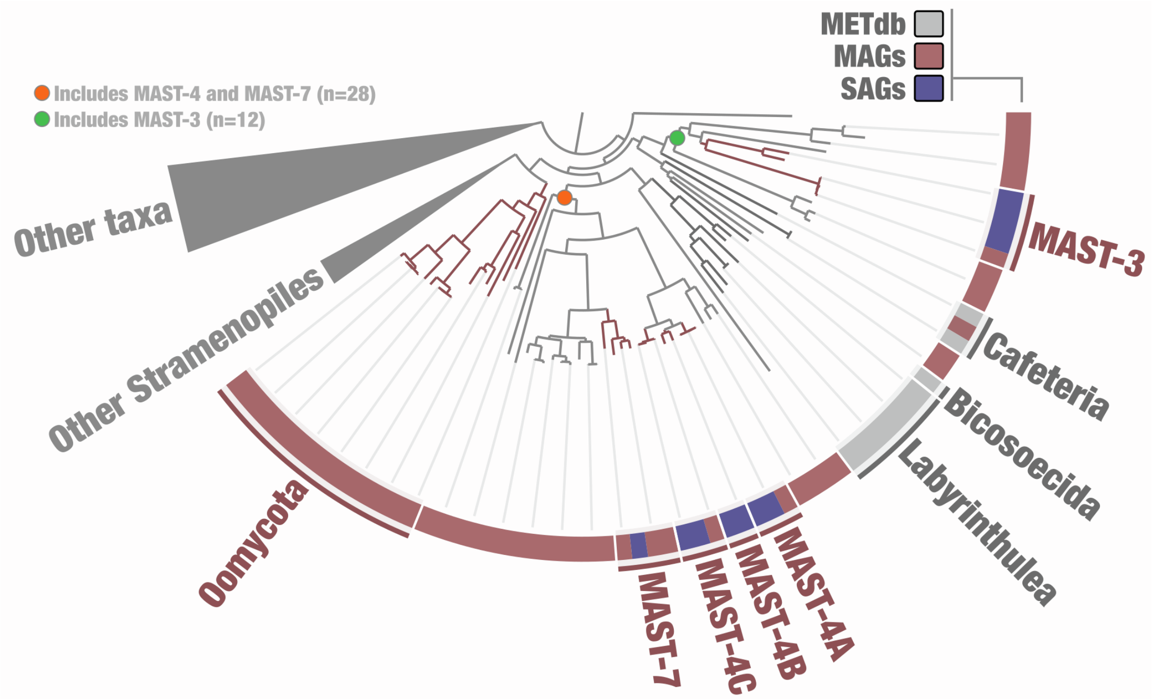

Figure S7: Phylogenetic analysis of concatenated DNA-dependent RNA polymerase protein sequences from eukaryotic plankton, Related to Figure 2. The maximum-likelihood phylogenetic tree of the concatenated two largest subunits from the three DNA-dependent RNA polymerases (six genes in total) included *Tara* Oceans MAGs and SAGs along with METdb transcriptomes and was generated using a total of 7,243 sites in the alignment and LG+F+R10 model; Here large groups were collapsed to better visualize the diversity of MAST lineages. SAGs were affiliated to taxonomic lineages based on 18S rRNA gene analyses.

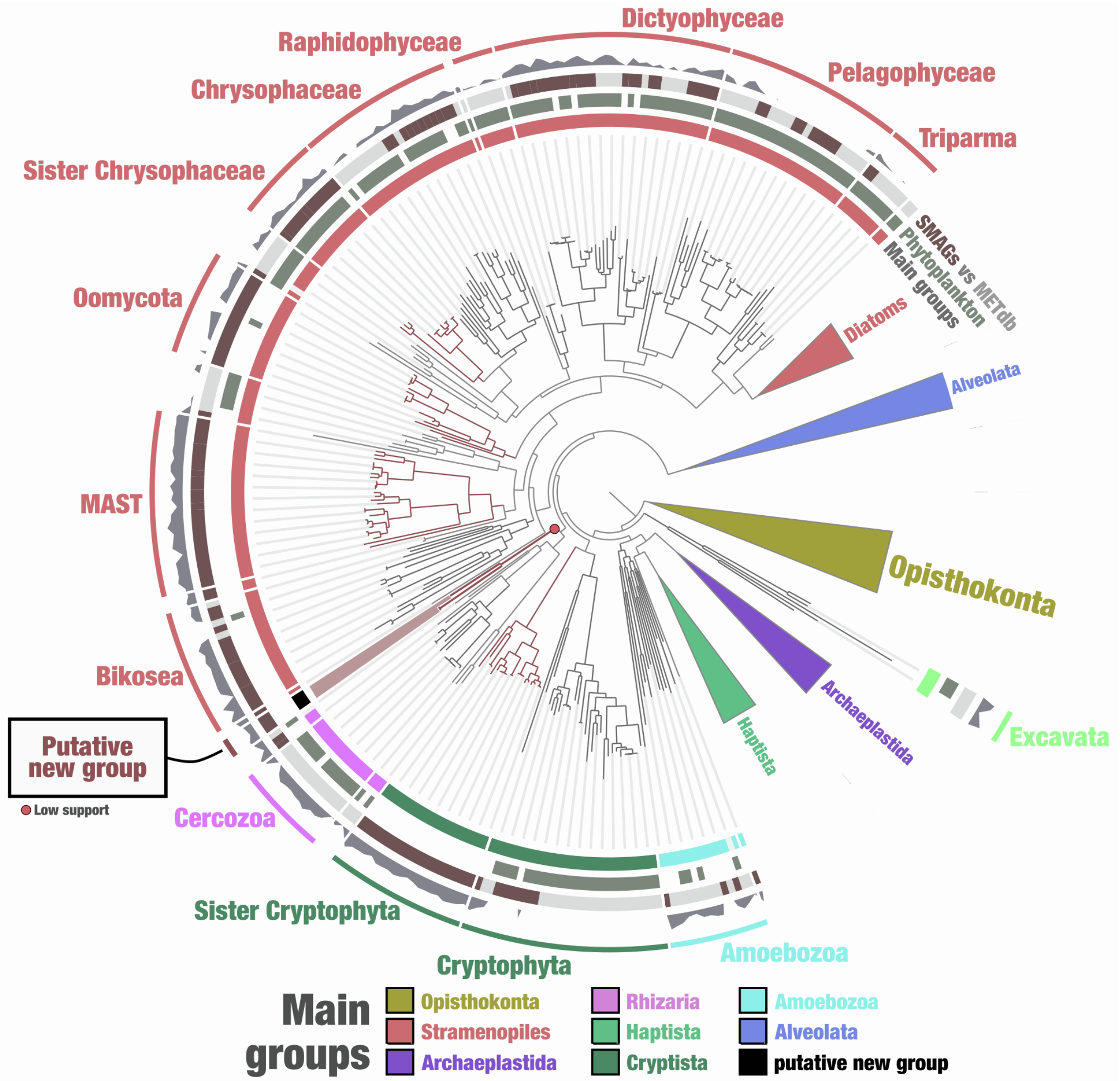

Figure S8: Phylogenetic analysis of concatenated DNA-dependent RNA polymerase protein sequences from eukaryotic plankton, Related to Figure 2. The maximum-likelihood phylogenetic tree of the concatenated two largest subunits from the three DNA-dependent RNA polymerases (six genes in total) included *Tara* Oceans MAGs and SAGs along with METdb transcriptomes and was generated using a total of 7,243 sites in the alignment and LG+F+R10 model; Opisthokonta was used as the outgroup. Support for the putative new group is displayed. Phylogenetic supports were considered high (aLRT>=80 and UFBoot>=95), medium (aLRT>=80 or UFBoot>=95) or low (aLRT<80 and UFBoot<95) (see Methods).

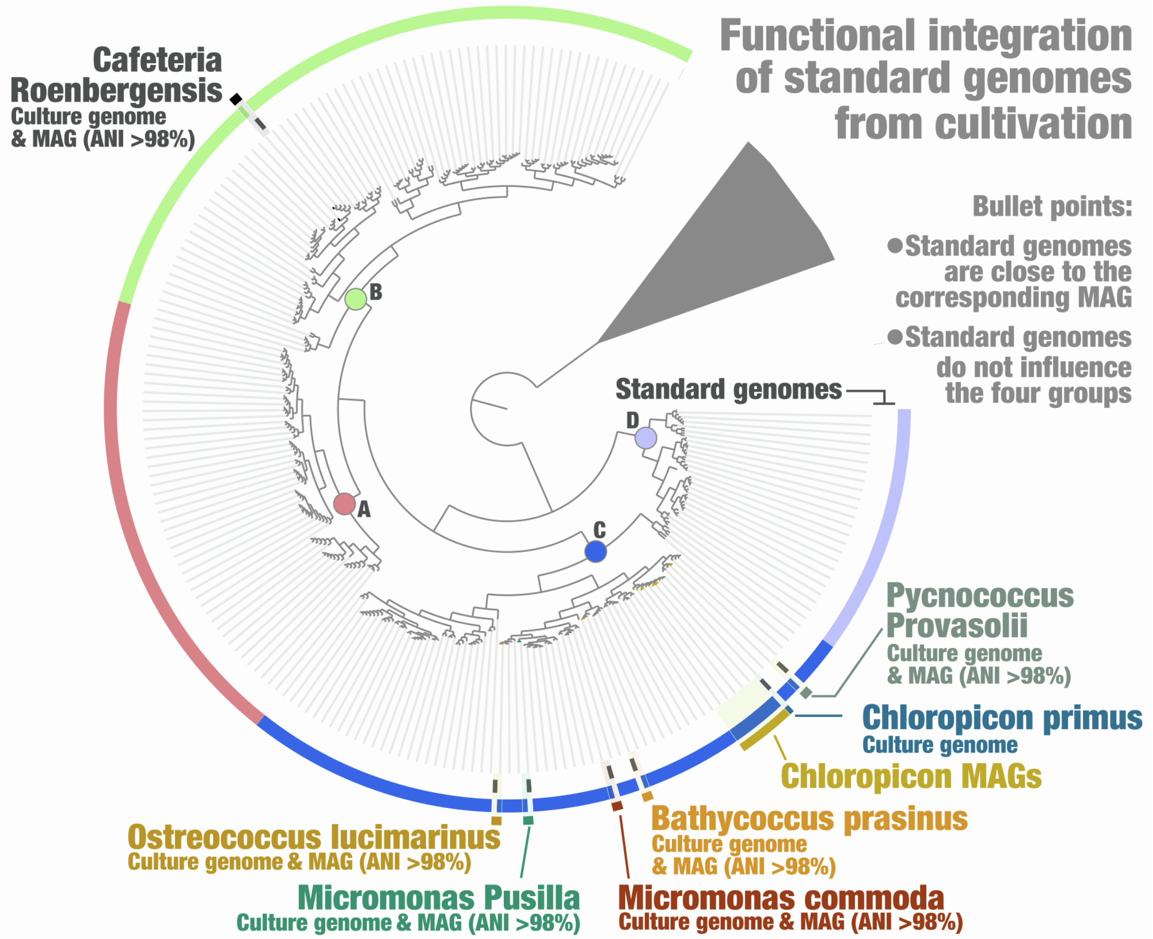

Figure S9: Functional clustering based on MAGs, SAGs and seven culture genomes, Related to Figure 3. The figure displays a hierarchical clustering (Euclidean distance with Ward's linkage) of MAGs, SAGs and seven closely related standard culture genomes (predicted proteins were imported from NCBI) based on the occurrence of the functions identified with EggNOG, rooted with small animals (Chordata, Crustacea and copepods) and decorated with layers of information using the anvi'o interactive interface. As for the previous analyses, removed from the analyses were Ciliophora MAGs (gene calling is problematic for this lineage), and functions occurring more than 500 times in the gigabase-scale MAG and linked to retrotransposons connecting otherwise unrelated MAGs and SAGs.

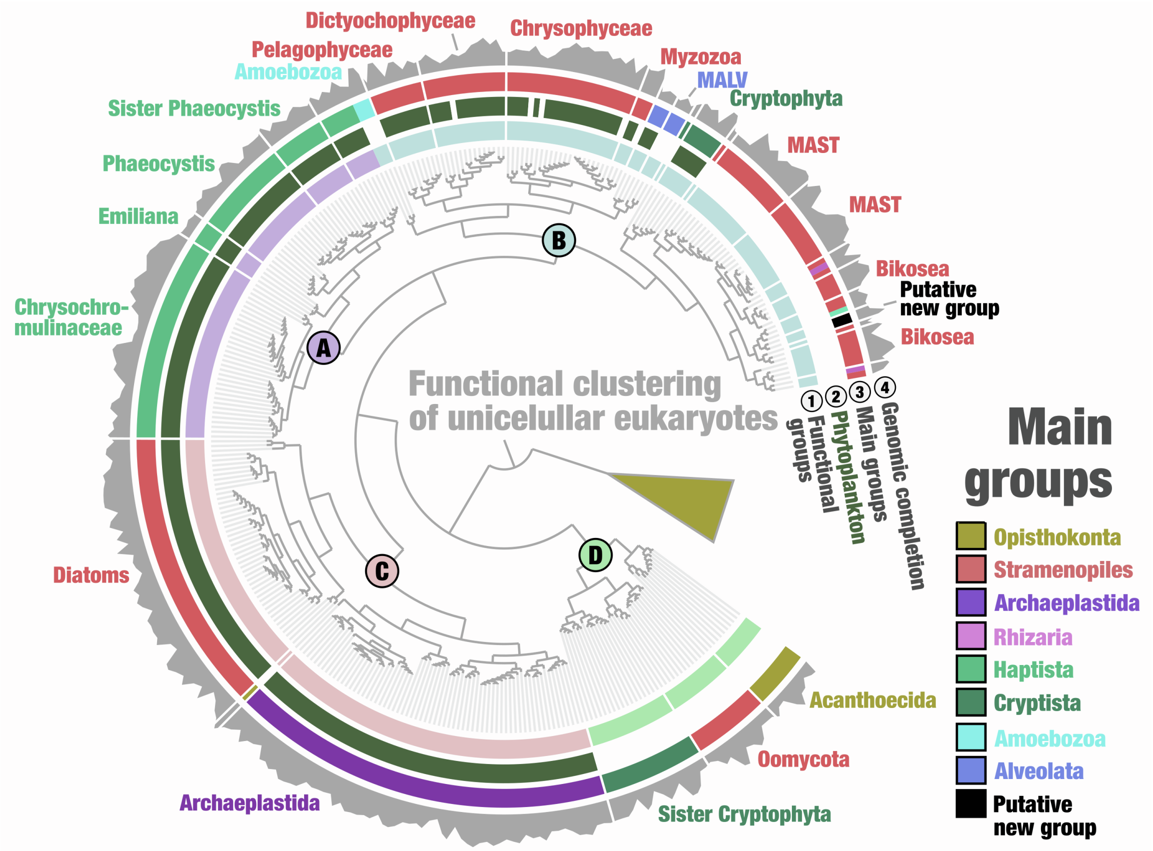

Figure S10: Functional clustering based on MAGs and SAGs with high completion estimates, Related to Figure 3. The figure displays a hierarchical clustering (Euclidean distance with Ward's linkage) of 483 MAGs and SAGs >25% complete (BUSCO estimation) based on the occurrence of 27,415 functions identified with EggNOG, rooted with small animals (Chordata, Crustacea and copepods) and decorated with layers of information using the anvi'o interactive interface. As for the previous analyses, removed from the analyses were Ciliophora MAGs (gene calling is problematic for this lineage), and functions occurring more than 500 times in the gigabase-scale MAG and linked to retrotransposons connecting otherwise unrelated MAGs and SAGs.

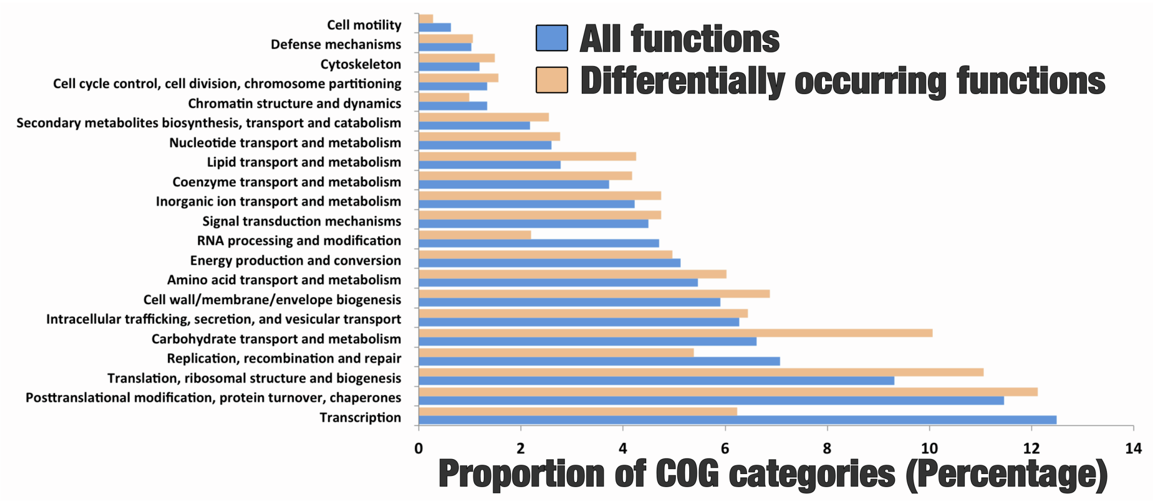

Figure S11. Relative proportion of known COG categories in annotated functions versus those that were significantly differentially occurring between the four functional groups, Related to Figure 3.

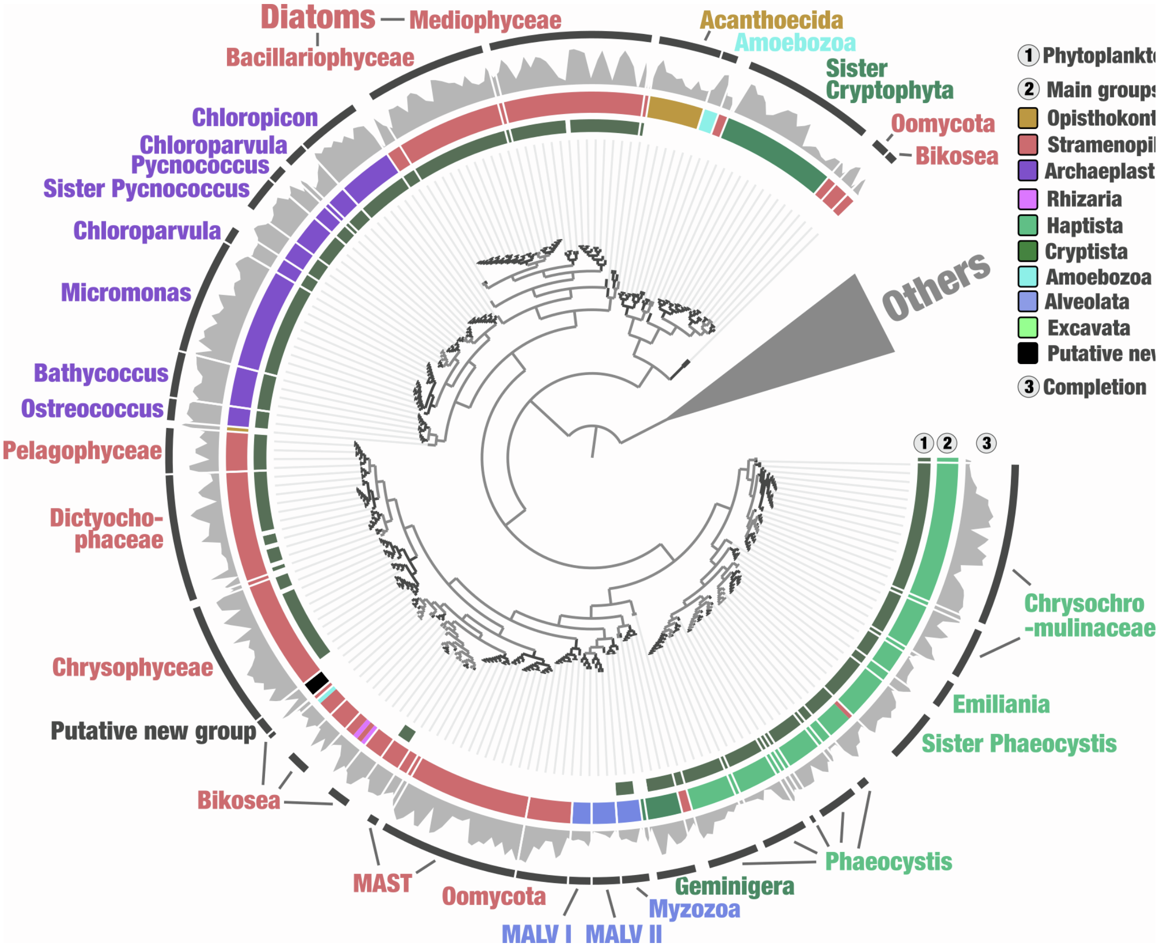

Figure S12. Functional landscape of unicellular eukaryotes in the sunlit ocean by combining EggNOG and Agnostos for gene processing, Related to Figure 3. The figure displays a hierarchical clustering (Euclidean distance with Ward's linkage) of 681 MAGs and SAGs based on the occurrence of ~39,705 groups of genes (total of 5,178,829 genes) identified by combining EggNOG[58–60] with Agnostos[65], rooted with MAGs dominated by small animals (Chordata, Crustacea and copepods) and decorated with layers of information using the anvi'o interactive interface. Removed from the analysis were Ciliophora MAGs (gene calling is problematic for this lineage), and functions occurring more than 1,000 times in the gigabase-scale MAG and linked to retrotransposons connecting otherwise unrelated MAGs and SAGs, or occurring in less than 2% of the MAGs and SAGs.

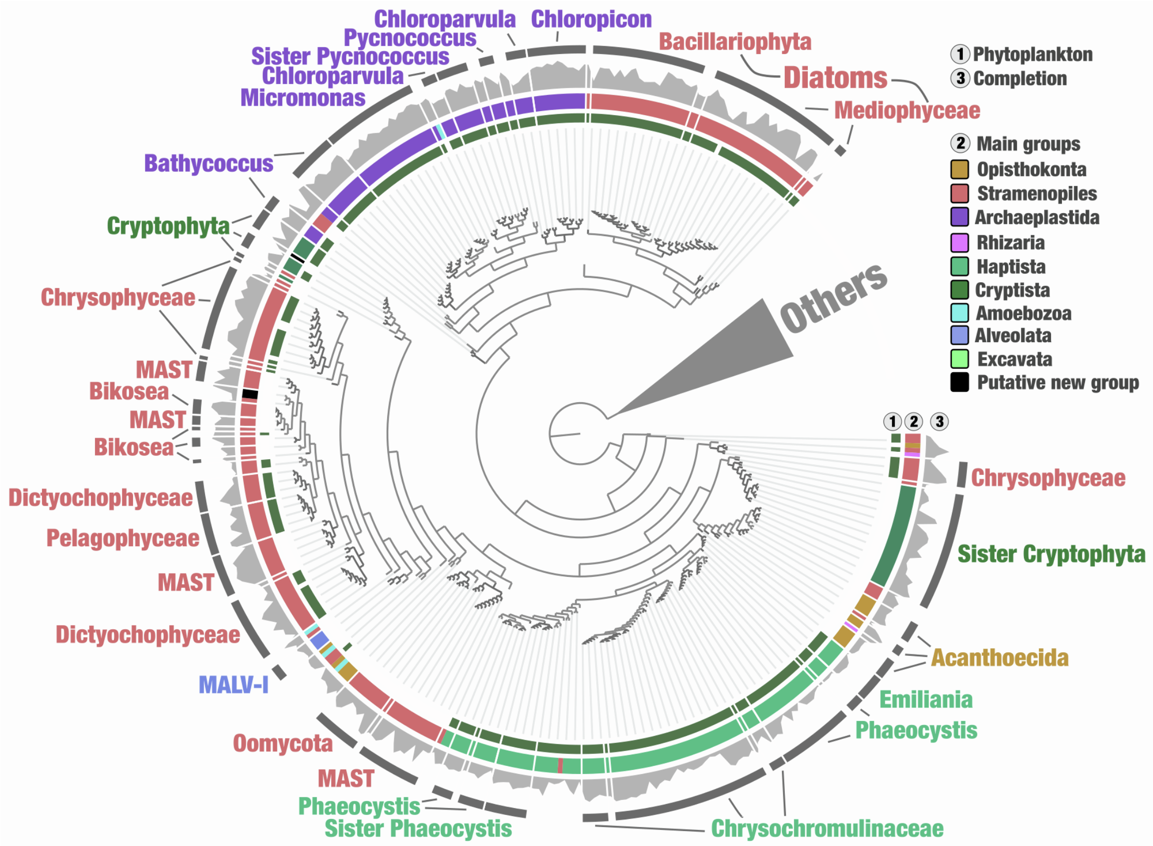

Figure S13. The genomic unknown functional landscape of unicellular eukaryotes in the sunlit ocean, Related to Figure 3. The figure displays a hierarchical clustering (Euclidean distance with Ward's linkage) of 681 MAGs and SAGs based on the occurrence of ~28,000 gene clusters of unknown function (total of 1.3 million genes) identified by solely with Agnostos[65] (environmental unknowns plus genomic unknowns), rooted with MAGs dominated by small animals (Chordata, Crustacea and copepods) and decorated with layers of information using the anvi'o interactive interface. Removed from the analysis were Ciliophora MAGs (gene calling is problematic for this lineage), and functions occurring more than 1,000 times in the gigabase-scale MAG and linked to retrotransposons connecting otherwise unrelated MAGs and SAGs, or occurring in less than 2% of the MAGs and SAGs.

Supplemental tables

Table S1.

Table S2.

Table S3.

Table S4.

Table S5.

Table S6.

Table S7.

Table S8.

Table S9.

Methods S1: supplemental methods, related to the STAR Methods.

#1: Genome-resolved metagenomics with anvi'o.

A set of single copy core genes to identify eukaryotic MAGs

As initially outlined in a blog post published at the beginning of this project to benefit others[1], we have defined a set of 83 single copy core genes from BUSCO[2] compatible with the gene calling workflow of anvi'o[3] to best estimate the completion of eukaryotic metagenome-assembled genomes (MAGs). Figure S14 describes the efficacy of this collection to estimate completion of MAGs from *Micromonas* and *Ostreococcus*. Note that those estimates are only initial, since this stage of the workflow uses a gene calling (Prodigal[4]) that is not optimal for eukaryotes. However, the results are sufficiently robust to effectively guide the manual binning and curation of eukaryotic MAGs without the need to first identify eukaryotic contigs in the assembly output. While the identification of eukaryotic contigs prior to binning as been benchmarked by the group of Jill banfield[5], false positives and false negatives associated with this critical step can be problematic and are entirely avoided in our workflow. We found that binning metagenomes containing multiple domains of life can be done smoothly within anvi'o, as long as proper single copy core gene collections are used to efficiently affiliate MAGs to Bacteria, Archaea and Eukarya. Note that this dedicated collection for eukaryotes is the main improvement within anvi'o compared to the workflow outlined for the characterization of ~1,000 bacterial and archaeal MAGs from small size fractions of TARA Oceans[6]. It is now an integral component of the anvi'o metagenomic flow used by a growing number of scientists interested in genome-resolved metagenomics.

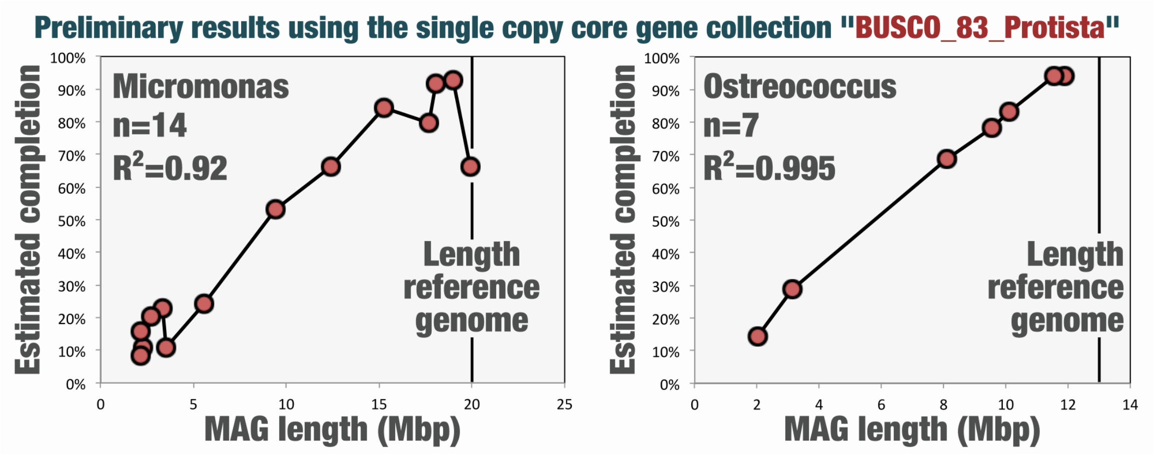

Figure S14: Completion estimates for *Micromonas* and *Ostreococcus* MAGs using a set of 83 BUSCO single copy core genes, as a function of the length of the MAGs.

A summary of the workflow to bin and curate eukaryotic MAGs

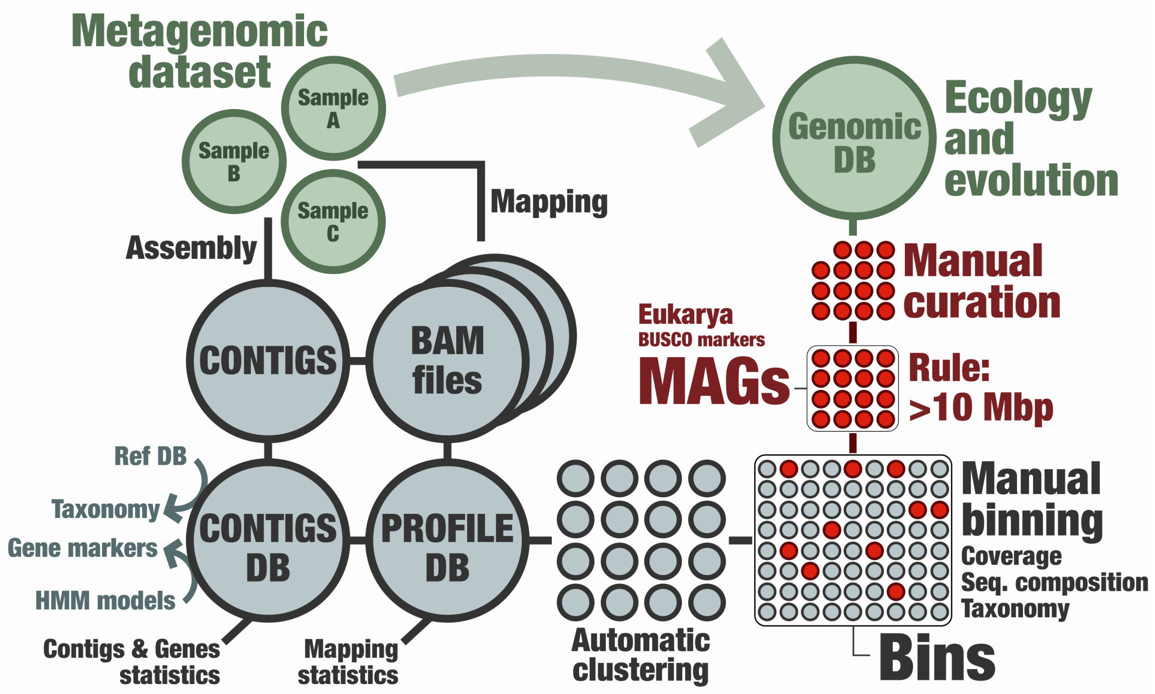

Figure S15: The manual genome-resolved metagenomic framework of anvi'o dedicated to the eukaryotes. This workflow is to be applied to each assembly outcome.

We followed the workflow outlined in the figure s15 for each of the 11 metagenomic co-assemblies outlined in the study (see Table S2). Briefly, we used the sequence composition of contigs and their differential coverage across metagenomes to perform a first automatic binning step with CONCOCT[7] by constraining the number of created clusters (thereafter dubbed metabins) to a number substantially below the number of genomes in the assembly. This number ranged from 50 to 400 depending on the assembly volume. Note that CONCOCT is used because the interactive interface of anvi'o cannot work efficiently when loading >25k contigs.

For each metabin, we then used the anvi'o interactive interface to manually identify and curate eukaryotic MAGs. This step took about 10 months of manual work.

An holistic interactive interface now compatible with eukaryotes

Within the framework of our study, the anvi'o interactive interface took advantage of the sequence composition of contigs, their differential coverage across metagenomes, taxonomic signal using a reference database that includes METdb, and HMM models for single copy core gene collections (Bacteria, Archaea, Eukarya). When selecting a cluster of contigs corresponding to a MAG in the interface, anvi'o identified its domain affiliation in real time using random forest, and displayed its completion and redundancy values accordingly. This way, it was possible to focus on the eukaryotic MAGs within an assembly containing also many abundant bacterial and archaeal MAGs. In the figure S16, we provide the example of one metabin from the Mediterranean Sea metagenomic co-assembly (95 metagenomes) containing eukaryotic MAGs for *Ostreococcus* and *Micromonas* (left panel). In this simple example, we selected those two clusters in the interface, saved the collection, and subsequently manually curated them as presented here for *Ostreococcus* (right panel). This MAG exhibited a completion of 100% and a redundancy of 3%. One metagenome (most outer blue layer) was particularly useful in this particular case since the *Micromonas* MAG was more detected compared to the Ostreococcus MAG, allowing an effective binning outcome. Given the complexity of marine metagenomes, differential coverage across dozens of metagenomes strongly benefited to the outcome of our genome-resolved metagenomic survey.

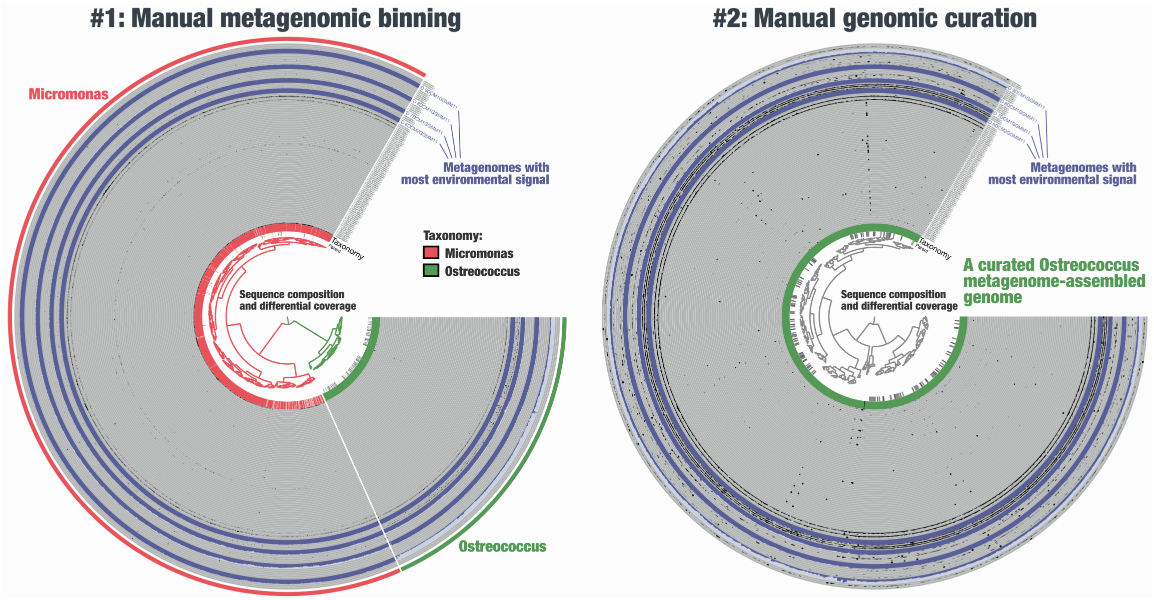

Figure S16: The anvi'o interactive interface to manually bin and curate eukaryotic MAGs. The left panel displays the detection of contigs from a single metabin across 95 metagenomes, alongside taxonomic signal. Clustering was done using sequence composition and differential coverage. Right panel displays the curated *Ostreococcus* MAGs identified from the left panel.

Example of environmental signal for a manually curated Ciliophora MAG

We provide an example of manually curated MAG ("TARA MED 95 MAG 00445"), for which environmental signal is described using both detection (horizontal coverage, left panel) and mean coverage (vertical coverage, right panel):

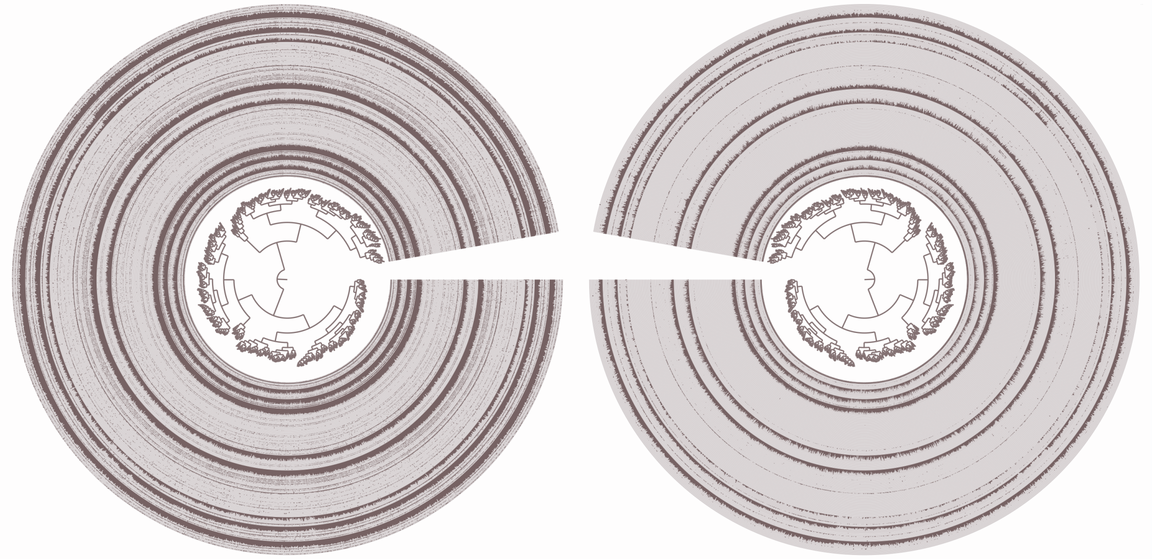

Figure S17: Example of a manually curated eukaryotic MAG as visualized in the anvi'o interactive. The selected MAG is named "TARA MED 95 MAG 00445". It is affiliated to Ciliophora and contains 2,613 contigs for a length of 13.5 Mbp. Clustering of contigs was done using sequence composition alone. Then, the left and right panels respectively display the detection and mean coverage of contigs across the 95 *Tara* Oceans metagenomes, which were used to access the quality of this MAG and others.

We can observe strong environmental signal coherence for the 2,614 contigs. The contigs correlated across the 95 metagenomes considered, with no particular outliers when it comes to sequence composition either. Critically, the coherence of environmental signal is supportive of the quality of the MAGs, which were all manually inspected and curated.

#2: Decontamination of single cell genomes with anvi'o.

Eukaryotic single cell genomes (SAGs) can be heavily contaminated due to a combination of factors during cell sorting, DNA extraction and amplification, and multiplex sequencing. Here, we slightly modified the anvi'o metagenomic workflow to effectively decontaminate marine eukaryotic SAGs, one by one. Briefly, we used the anvi'o interactive interface to manually curate eukaryotic SAGs by taking into consideration the sequence composition of contigs, their differential coverage across 100 most relevant metagenomes (i.e., those with highest mapping recruitment scores within the scope of TARA Oceans), taxonomic signal using a reference database that includes METdb, and HMM models for single copy core gene collections (Bacteria, Archaea, Eukarya). Note that compared to the metagenomic co-assemblies, the number of contigs under consideration was orders of magnitude

smaller. Since all contigs could be loaded in the interactive interface, there was no need to use the pre-clustering step with CONCOCT. However, CONCOCT could also be used here if some SAG assemblies include more than ~25k contigs.

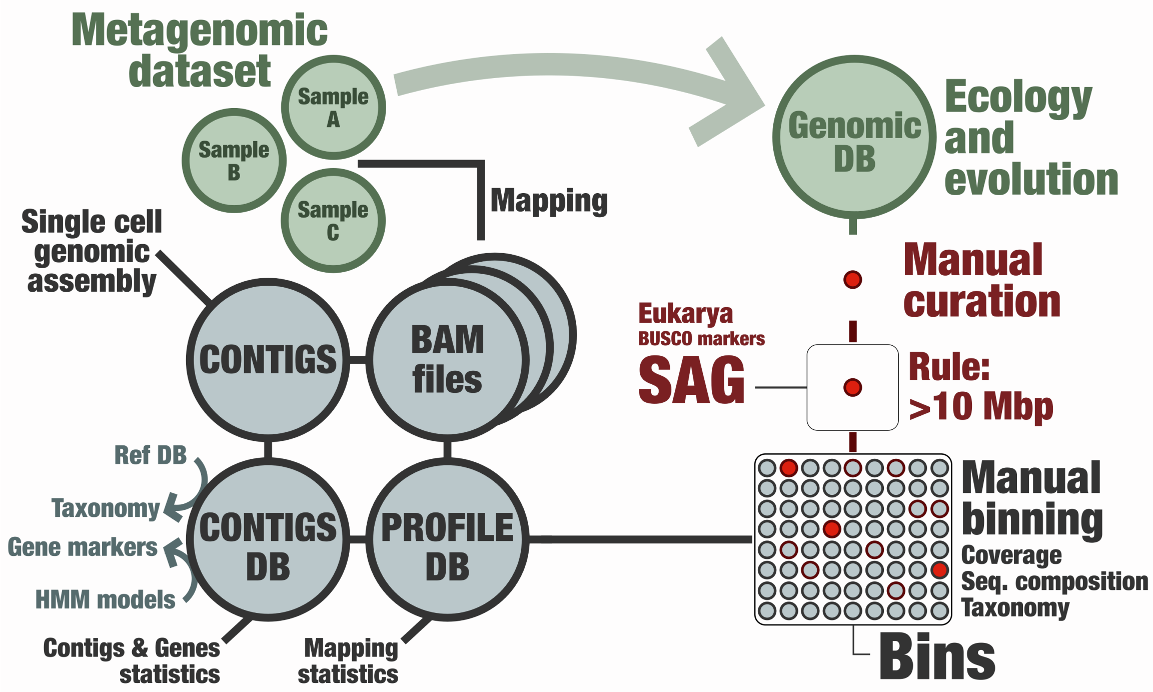

Figure S18: The manual metagenomic framework of anvi'o dedicated to the decontamination of SAGs. This workflow was applied to each SAG (co-)assembly outcome.

Figure S19 provides a striking example of heavily contaminated SAG we could effectively curate thanks to the clear differential coverage signal of contigs across 100 metagenomes. In this particular case, contamination seemed to have multiple origins, and a large number of contigs were removed. Overall, our manual curation of SAGs using a genome-resolved metagenomics workflow initially built for MAGs turned out to be highly valuable, leading in our study to the removal of more than one hundred thousand scaffolds for a total volume of 193.1 million nucleotides. This metagenomic-guided decontamination effort contributes to previous efforts characterizing eukaryotic SAGs from the same cell sorting material[8–12] and provides new guidelines for marine eukaryotic SAGs. We now recommend this approach for future efforts generating eukaryotic SAGs from the sunlit ocean. This is important, especially since SAGs could become a valuable asset in the near future to target lineages genome-resolved metagenomics failed to recover so far. It is especially the case of Dinoflagellates.

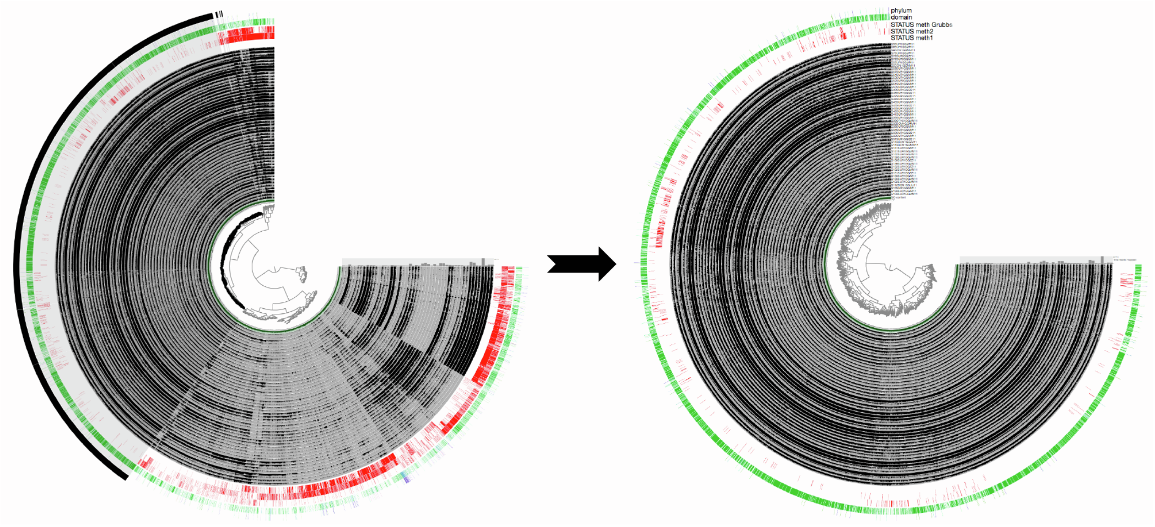

Figure S19: Example of the decontamination of TOSAG00-8. Left panel describes all contigs reconstructed from this SAG, organized based on sequence composition and differential coverage across 100 *Tara* Oceans metagenomes. The selection of contigs (outer layer) corresponds to our final curated SAG, displayed in the right panel, for which clustering is base don sequence composition alone.

#3: The METdb database for eukaryotic transcriptomes.

METdb is a curated database of transcriptomes from marine eukaryotic isolates that cover the MMETSP collection[13] (new assemblies were performed, combining time points from the same culture in co-assemblies when available) as well as cultures from TARA Oceans. The associated manuscript is not yet published. However, the database is publically available and can be accessed at http://metdb.sb-roscoff.fr/metdb/.

#4: World map projections.

World Ocean Atlas data

Seven physicochemical parameters were used to define environmental niches: sea surface temperature (SST), salinity (Sal), dissolved silica (Si), nitrate (NO_3), phosphate (PO_4), iron (Fe), and a seasonality index of nitrate (SI NO_3). With the exception of Fe and SI NO3, these parameters were extracted from the gridded World Ocean Atlas 2013 (WOA13)[21]. Climatological Fe fields were provided by the biogeochemical model PISCES-v2[22]. The seasonality index of nitrate was defined as the range of nitrate concentration in one grid cell divided by the maximum range encountered in WOA13 at the Tara sampling stations. All parameters were co-located with the corresponding stations and extracted at the month corresponding to the Tara sampling. To compensate for missing physicochemical samples in the Tara *in situ* data set, climatological data (WOA) were favored. The correlation between *in situ* samples and corresponding values extracted from WOA were high:

R-squared values for the surface samples:
SST: 0.99, Sal: 0.86, Si: 0.89, NO3: 0.85, PO4: 0.90

R-squared values for the DCM samples:
SST: 0.97, Sal: 0.47, Si: 0.97, NO3: 0.74, PO4: 0.85

In the absence of corresponding WOA data, a search was done within 2° around the sampling location and values found within this square were averaged.

Nutrients, such as NO_3 and PO_4, displayed a strong collinearity when averaged over the global ocean (correlation of 0.95 in WOA13), which could complicate disentangling their respective contribution to niche definition. However, observations and experimental data allow distinguishing between limiting nutrients at regional scale characterized by specific plankton communities[23]. The future projection of niches will yield spurious results when the present-day collinearity is not maintained[24,25]. To this day, there is no evidence for large scale changes in global nutrient stoichiometry[26].

Earth System Models and bias correction

Outputs from six Earth system models were used to project environmental niches under greenhouse gas emission scenario RCP8.5[27]:

Model	Reference
CESM1-BGC	Gent et al., 2011
GFDL-ESM2G	Dunne et al., 2013
GFDL-ESM2M	Dunne et al., 2013
HadGEM2-ES	Collins et al., 2011
IPSL-CM5A-LR	Dufresne et al., 2013
IPSL-CM5A-MR	Dufresne et al., 2013
MPI-ESM-LR	Giorgetta et al., 2013
MPI-ESM-MR	Giorgetta et al., 2013
NorESM1-ME	Bentsen et al., 2013

Table 1: Summary of Earth system models used to project environmental niches.

Environmental drivers were extracted for present day (2006-2015) and end of century (2090-2099) conditions for each model and the multi-model mean was computed. A bias correction method, the Cumulative Distribution Function transform, CDFt[28], was applied to adjust the distributions of SST, Sal, Si, NO_3 and PO_4 of the multi-model mean to the WOA database. CDFt is based on a quantile mapping (QM) approach to reduce the bias between modeled and observed data, while accounting for climate change. Therefore, CDFt does not rely on the stationary hypothesis and present and future distributions can be different. CDFt was applied on the global fields of the mean model simulations. By construction, CDFt preserved the ranks of the simulations to be corrected. Thus, the spatial structures of the model fields were preserved.

Environmental niches models: training, validation and projections

From the initial dataset of 713 SMAGs, we selected those present in at least 4 stations for environmental niche training, discarding just 58 of them. Four machine learning methods were applied to compute environmental niches for each of the 655 remaining SMAGs:

(1) Gradient Boosting Machine (gbm)[29]
(2) Random Forest (rf)[30]
(3) Fully connected Neural Networks (nn)[31]
(4) Generalized Additive Models (gam)[32]

Hyper parameters of each technique (except gam) were optimized as followed:

(1) For gbm, the interaction depth (1, 3 and 5), learning rate (0.01, 0.001) and the minimum number of observations in a tree node (1 to 10)
(2) For rf, the number of trees (100 to 900 with step 200 and 1000 to 9000 with step 2000) and the number of parameters used for each tree (1 to 8)
(3) For nn, the number of layers of the network (1 to 10) and the decay (1.10-4 to 9.10-4 and 1.10-5 to 9.10-5)
(4) For gam the number of splines was set to 3.

R packages gbm (2.1.3), randomForest (4.6.14), mgcv (1.8.16) and nnet (7.3.12) were used for gbm, rf, nn and gam models.

To define the best combination of hyper parameters for each model, we perform 30 random cross-validations by training the model on 75% of the dataset randomly sampled and by calculating the Area Under the Curve51 (AUC) on the 25% remaining points of the dataset. The best combination of hyper parameters is the one for which the mean AUC over the 30 cross-validation is the highest. A model is considered valid if at least 3 out of the 4 techniques have a mean AUC superior to 0.65, which is the case for 374 out of the 655 SMAGs (57%). Final models are trained on the full dataset and only the techniques that have a mean AUC higher than 0.65 are considered to make the projections. The majority (286) of the 374 validated niches is validated by all four models and 88 by only 3 models. Relative influences of each parameter in defining environmental niches are calculated using the feature_importance function from the DALEX R package[33] for all four statistical methods. For model training and projections, physicochemical variables are scaled to have a mean of 0 and a variance of 1. For this scaling, the mean and standard deviation of each WOA13 variable (+ PISCES-v2 Fe) co-localized with Tara stations with a value available is used. This standardization procedure allows for better performance of models. Finally, as statistical models often disagree we use the ensemble model approach for global-scale projections of niches[34] i.e. the mean projections of the validated machine learning techniques.

Environmental niches models at Tara Oceans stations

Here we describe the performances of the statistical models on biogeochemical model projections at locations of the training set (*i.e.* the Tara stations). Our models are only presence/absence models so they project probabilities of presence (not relative abundances) of a given MAG at each gridded point of the ocean based on environmental parameters. The figure S20 presents the specificity in function of the sensitivity for each model (*i.e.* each point is a MAG) calculated on the set of *Tara* stations for biogeochemical projections and for two threshold of presence detection (p>0.5 and p>0.3). The specificity captures the ability of the model to correctly detect absences while the sensitivity captures its capability to detect presences. Details on model computation and validation are in the supplementary material.

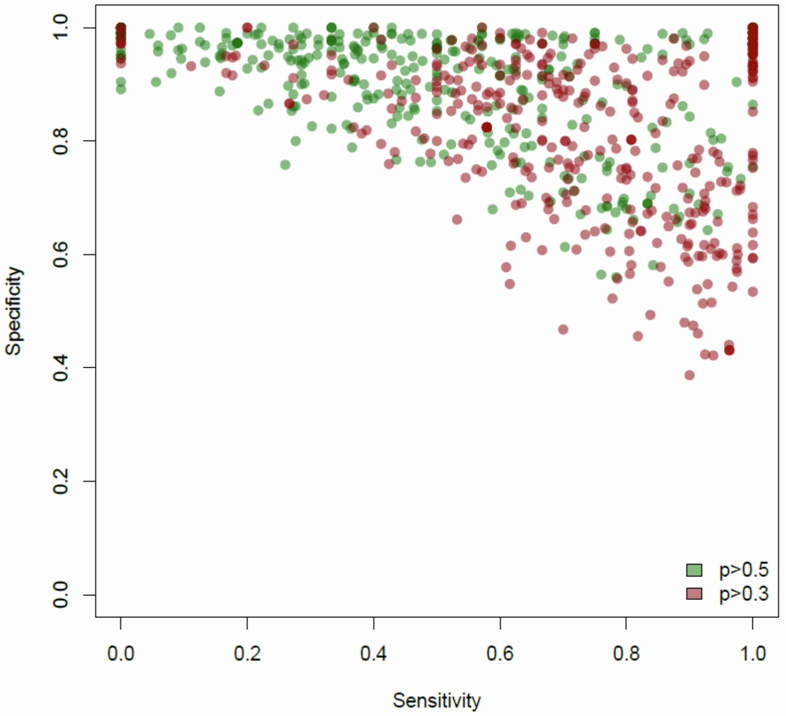

Figure S20: Statistical niches model performances for each MAG on biogeochemical model projections at locations of the training set (*Tara* stations). Specificity, *i.e.* the capacity of the model to correctly project absences is represented in function of sensitivity, *i.e.* the capacity of the model to correctly project presences for each MAG for which a valid environmental niche was found. Two thresholds for presence detection are used (in green p>0.5, in red p>0.3).

Globally, models perform well, especially for p>0.3 as a presence threshold, with a vast majority of models with sensitivity>0.6 and sensibility>0.6 (61% for p>0.3, 39% for p>0.5). Lowering the presence threshold allows a global increase in sensitivity with a relatively low decrease in specificity (red points versus green points). Some models perform relatively poorly and have low sensitivity. This might be explained by the asymmetry in number of presences compared to absences in the training set (relatively many more absences). In addition, the spatial structure and

resolution as well as the hidden seasonality (10 years climatologies are used) of the biogeochemical models might explain these discrepancies.

#5: Categorizing the 939 TARA Oceans metagenomes.

Our study surveyed a total of 939 TARA Oceans metagenomes (Table S1) that we organized into four cellular size categories (**size 1**: 0.2-5µm, **size 2**: 3-20µm, **size 3**: 20-200µm, **size 4**: 180-2000µm) as well as a wider cellular size fraction encompassing all categories considered in our study (**wider size**: 0.8-2000µm). The four cellular size categories were well represented across the five oceans and two seas. Overall, 119 stations contained at least 3 out of the 4 cellular size categories, which we defined as **Station subset 1** (757 metagenomes). Using this first subset, SMAGs were assigned a "**cosmopolitan score**" corresponding to the percentage of stations in which they were detected. SMAGs were also assigned a "**cellular size range"** and "**oceanic signal"** using average coverage in each size categories (n=4) for the former and in each ocean and sea (n=7) for the later. Those results are summarized in the tables S3 an S4. Unfortunately, the wider cellular size fraction was missing in the Mediterranean Sea, Red Sea and Indian Ocean, limiting its use to 91 stations from the four remaining oceans, which we defined as **Station subset 2** (130 metagenomes). Critically, this second subset offers a glimpse into the relative proportion of planktonic lineages of different cellular sizes. While more limited in its geographic scope, the **Station subset 2** could provide important insights into the **"relative proportion**" of SMAGs in stations from the Atlantic, Pacific, Arctic and Southern Ocean.

#6: Manual curation of the DNA-dependent RNA polymerase genes for SMAGs and METdb.

An eukaryotic dataset (Da Cunha)[14] was used to build HMM profiles for the two largest subunits of the DNA-dependent RNA polymerase (RNAP-a and RNAP-b). These two HMM profiles were incorporated within the anvi'o framework to identify RNAP-a and RNAP-b genes (Prodigal[4] annotation) in the SMAGs and METdb transcriptomes.

We independently performed the following workflow for RNAP-a sequences identified in the SMAGs (round A, n= 1,626) and METdb (round B, n= 2,823) as well as for RNAP-b sequences identified in the SMAGs (round C, n= 1,373) and METdb (round D, n= 3,941):

(1) **Stetting the stage with references:** Reference sequences for the relevant largest subunits of the DNA-dependent RNA polymerase (e.g., RNAP-a for round A) corresponding to eukaryotic (types I, II and III), bacterial and archaeal lineages from the Da Cunha dataset were added to the sequences identified by the HMM.

(2) **Phylogenetic tree Phase 1:** Sequences were aligned using the iterative FFT-NS-i refinement method of MAFFT[15] v7.464 with default parameters, and the sites with more than 50% of gaps were trimmed using Goalign v0.3.0-alpha5. Phylogenetic trees were reconstructed with IQ-TREE[16] v1.6.12. The model of evolution was estimated with the ModelFinder Plus option[17], and supports were computed from 1,000 replicates for the Shimodaira-Hasegawa (SH)-like approximation likelihood ratio (aLRT)[18] and ultrafast bootstrap approximation (UFBoot)[19]. Anvi'o v6.1 was used to visualize and root the phylogenetic trees.

(3) **Identifying sequences of type I, II and III:** We used the anvi'o interactive interface to root the tree between Bacteria and the rest, and identify sequences corresponding to eukaryotic DNA-dependent RNA polymerase of type I, II and III. Sequences not clearly belonging to one of these three clusters were discarded. Note that during this process other types of eukaryotic RNA polymerase (e.g., nucleomorphs) were identified and put aside for investigations beyond the scope of this study.

(4) **Fusing fragmented sequences when needed:** For each SMAG or METdb transcriptome, sequences corresponding to the same RNA polymerase type (e.g., RNAP-a_type_I for round A) were aligned against each other and against a relevant eukaryotic reference sequence using blastp[20]. Non-overlapping sequences corresponding to the same subunit (based on **Phylogenetic tree Phase 1**) were considered fragments of the same gene and fused manually, overcoming fragmentation issues during gene calling and/or transcription. In addition, only the longest sequence was kept for overlapping isoforms and closely related duplicates (>95% identity and >30% coverage).

(5) **Phylogenetic tree Phase 2:** A phylogenetic tree was performed for each subunit (DNA-dependent RNA polymerase of type I, II and III) as done for the **Phylogenetic tree Phase 1** (for improved resolution, archaeal references were used as outgroup and bacterial sequences removed in this analysis). Distantly related duplicates (those occurred in <5% of SMAGs and <10% of METdb transcriptomes, possibly due to contamination) were carefully considered in the context of the three phylogenetic trees as well as taxonomy to identify and remove sequences with incoherent phylogenetic and/or taxonomic signal.

(6) **Final collection:** We removed sequences shorter than 200 amino-acids, providing a final collection of DNA-dependent RNA polymerase genes for the SMAGs (n=2,150) and METdb (n=2,032) with no duplicates.

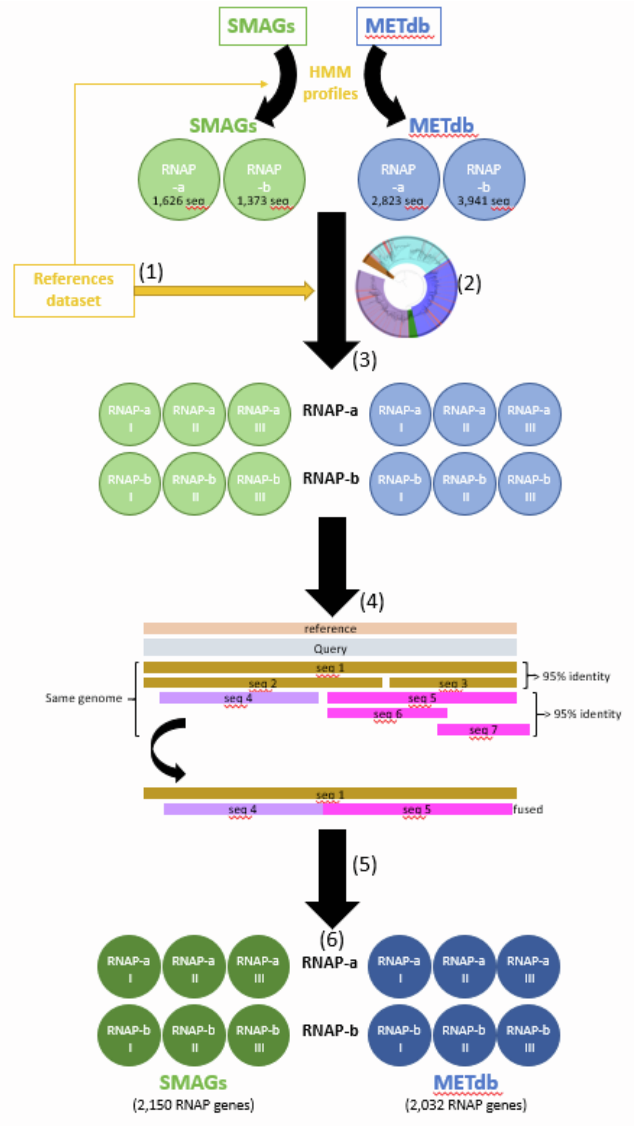

Figure S21: Workflow for the manual curation of RNA polymerase genes identified in the MAGs, SAGs and METdb culture transcriptomes.

References

1. Delmont, T.O. (2018). Assessing the completion of eukaryotic bins with anvi'o. Blog post. http://merenlab.org/2018/05/05/eukaryotic-single-copy-core-genes/.
2. Simão, F.A., Waterhouse, R.M., Ioannidis, P., Kriventseva, E. V., and Zdobnov, E.M. (2015). BUSCO: assessing genome assembly and annotation completeness with single-copy orthologs. Bioinformatics *31*, 3210–3212.
3. Eren, A.M., Esen, Ö.C., Quince, C., Vineis, J.H., Morrison, H.G., Sogin, M.L., and Delmont, T.O. (2015). Anvi'o: an advanced analysis and visualization platform for 'omics data. PeerJ *3*, e1319.
4. Hyatt, D., Chen, G.-L., Locascio, P.F., Land, M.L., Larimer, F.W., and Hauser, L.J.

(2010). Prodigal: prokaryotic gene recognition and translation initiation site identification. BMC Bioinformatics *11*, 119.

5. West, P.T., Probst, A.J., Grigoriev, I. V., Thomas, B.C., and Banfield, J.F. (2018). Genome-reconstruction for eukaryotes from complex natural microbial communities. Genome Res. *28*, gr.228429.117.
6. Delmont, T.O., Quince, C., Shaiber, A., Esen, Ö.C., Lee, S.T., Rappé, M.S., MacLellan, S.L., Lücker, S., and Eren, A.M. (2018). Nitrogen-fixing populations of Planctomycetes and Proteobacteria are abundant in surface ocean metagenomes. Nat. Microbiol. 2018 37 *3*, 804–813.
7. Alneberg, J., Bjarnason, B.S., de Bruijn, I., Schirmer, M., Quick, J., Ijaz, U.Z., Lahti, L., Loman, N.J., Andersson, A.F., and Quince, C. (2014). Binning metagenomic contigs by coverage and composition. Nat. Methods *11*, 1144–1146.
8. Sieracki, M.E., Poulton, N.J., Jaillon, O., Wincker, P., de Vargas, C., Rubinat-Ripoll, L., Stepanauskas, R., Logares, R., and Massana, R. (2019). Single cell genomics yields a wide diversity of small planktonic protists across major ocean ecosystems. Sci. Reports 2019 91 *9*, 1–11.
9. Seeleuthner, Y., Mondy, S., Lombard, V., Carradec, Q., Pelletier, E., Wessner, M., Leconte, J., Mangot, J.F., Poulain, J., Labadie, K., et al. (2018). Single-cell genomics of multiple uncultured stramenopiles reveals underestimated functional diversity across oceans. Nat. Commun. 2018 91 *9*, 1–10.
10. Mangot, J.F., Logares, R., Sánchez, P., Latorre, F., Seeleuthner, Y., Mondy, S., Sieracki, M.E., Jaillon, O., Wincker, P., Vargas, C. De, et al. (2017). Accessing the genomic information of unculturable oceanic picoeukaryotes by combining multiple single cells. Sci. Reports 2017 71 *7*, 1–12.
11. López-Escardó, D., Grau-Bové, X., Guillaumet-Adkins, A., Gut, M., Sieracki, M.E., and Ruiz-Trillo, I. (2017). Evaluation of single-cell genomics to address evolutionary questions using three SAGs of the choanoflagellate Monosiga brevicollis. Sci. Reports 2017 71 *7*, 1–14.
12. Vannier, T., Leconte, J., Seeleuthner, Y., Mondy, S., Pelletier, E., Aury, J.M., De Vargas, C., Sieracki, M., Iudicone, D., Vaulot, D., et al. (2016). Survey of the green picoalga Bathycoccus genomes in the global ocean. Sci. Reports 2016 61 *6*, 1–11.
13. Keeling, P.J., Burki, F., Wilcox, H.M., Allam, B., Allen, E.E., Amaral-Zettler, L.A., Armbrust, E.V., Archibald, J.M., Bharti, A.K., Bell, C.J., et al. (2014). The Marine Microbial Eukaryote Transcriptome Sequencing Project (MMETSP): Illuminating the Functional Diversity of Eukaryotic Life in the Oceans through Transcriptome Sequencing. PLOS Biol. *12*, e1001889.
14. Da Cunha, V., Gaia, M., Nasir, A., and Forterre, P. (2018). Asgard archaea do not close the debate about the universal tree of life topology. PLOS Genet. *14*, e1007215.
15. Katoh, K., and Standley, D.M. (2013). MAFFT Multiple Sequence Alignment Software Version 7: Improvements in Performance and Usability. Mol. Biol. Evol. *30*, 772–780.
16. Nguyen, L.T., Schmidt, H.A., Von Haeseler, A., and Minh, B.Q. (2015). IQ-TREE: A Fast and Effective Stochastic Algorithm for Estimating Maximum-Likelihood Phylogenies. Mol. Biol. Evol. *32*, 268–274.

17. Kalyaanamoorthy, S., Minh, B.Q., Wong, T.K.F., Von Haeseler, A., and Jermiin, L.S. (2017). ModelFinder: fast model selection for accurate phylogenetic estimates. Nat. Methods 2017 146 *14*, 587–589.
18. Guindon, S., Dufayard, J.F., Lefort, V., Anisimova, M., Hordijk, W., and Gascuel, O. (2010). New algorithms and methods to estimate maximum-likelihood phylogenies: Assessing the performance of PhyML 3.0. Syst. Biol. *59*, 307–321.
19. Hoang, D.T., Chernomor, O., Von Haeseler, A., Minh, B.Q., and Vinh, L.S. (2018). UFBoot2: Improving the Ultrafast Bootstrap Approximation. Mol. Biol. Evol. *35*, 518–522.
20. Altschul, S.F., Gish, W., Miller, W., Myers, E.W., and Lipman, D.J. (1990). Basic local alignment search tool. J. Mol. Biol. *215*, 403–410.
21. Boyer, T.P., Antonov, J.I., Baranova, O.K., Coleman, C., Garcia, H.E., Grodsky, A., Johnson, D.R., Locarnini, R. a, Mishonov, A. V, O'Brien, T.D., et al. (2013). WORLD OCEAN DATABASE 2013, NOAA Atlas NESDIS 72. Sydney Levitus, Ed.; Alexey Mishonoc, Tech. Ed.
22. Aumont, O., Ethé, C., Tagliabue, A., Bopp, L., and Gehlen, M. (2015). PISCES-v2: An ocean biogeochemical model for carbon and ecosystem studies. Geosci. Model Dev. *8*, 2465–2513.
23. Moore, C.M., Mills, M.M., Arrigo, K.R., Berman-Frank, I., Bopp, L., Boyd, P.W., Galbraith, E.D., Geider, R.J., Guieu, C., Jaccard, S.L., et al. (2013). Processes and patterns of oceanic nutrient limitation. Nat. Geosci *6*, 701–710.
24. Dormann, C.F., Elith, J., Bacher, S., Buchmann, C., Carl, G., Carré, G., Marquéz, J.R.G., Gruber, B., Lafourcade, B., Leitão, P.J., et al. (2013). Collinearity: a review of methods to deal with it and a simulation study evaluating their performance. Ecography (Cop.). *36*, 27–46.
25. Brun, P., Kiørboe, T., Licandro, P., and Payne, M.R. (2016). The predictive skill of species distribution models for plankton in a changing climate. Glob. Chang. Biol. *22*, 3170–3181.
26. Redfield, A.. (1934). On the Proportions of Organic Derivatives in Sea Water and Their Relation to the Composition of Plankton. James Johnstone Meml. Vol. Univ. Press Liverpool, 176–192. https://www.scirp.org/(S(351jmbntvnsjt1aadkposzje))/reference/ReferencesPapers.aspx?ReferenceID=1883475.
27. van Vuuren, D.P., Edmonds, J., Kainuma, M., Riahi, K., Thomson, A., Hibbard, K., Hurtt, G.C., Kram, T., Krey, V., Lamarque, J.F., et al. (2011). The representative concentration pathways: An overview. Clim. Change *109*, 5–31.
28. Michelangeli, P.A., Vrac, M., and Loukos, H. (2009). Probabilistic downscaling approaches: Application to wind cumulative distribution functions. Geophys. Res. Lett. *36*.
29. Ridgeway, G. (2006). Generalized boosted regression models. Doc. R Packag. "gbm", version.
30. Breiman, L., and Cutler, A. (2012). Breiman and Cutler's random forests for classification and regression. Packag. "randomForest."
31. Venables, W.N., and Ripley, B.D. (2002). Modern Applied Statistics with S Fourth edition by.
32. Wood, S.N. (2004). Stable and efficient multiple smoothing parameter

estimation for generalized additive models. J. Am. Stat. Assoc.
33. Biecek, P. (2018). Dalex: Explainers for complex predictive models in R. J. Mach. Learn. Res., 1−5.
34. Jones, M.C., and Cheung, W.W.L. (2015). Multi-model ensemble projections of climate change effects on global marine biodiversity. ICES J. Mar. Sci. *72*, 741–752.
